# An economic assessment of embryo diagnostics (Dx) - the costs of introducing non-invasive embryo diagnostics into IVF standard treatment practices

**DOI:** 10.1186/1472-6963-14-482

**Published:** 2014-10-09

**Authors:** Hans-Joerg Fugel, Mark Connolly, Mark Nuijten

**Affiliations:** Department of Pharmaoepidemiology and pharmacoeconomics, University of Groningen, 9713 Groningen, The Netherlands; Global Market Access Solutions, Health Economics, Charlotte, NC USA; Ars Accessus Medica BV, Dorpsstraat 75, Amsterdam (Jisp), The Netherlands

## Abstract

**Background:**

New techniques in assessing oocytes and embryo quality are currently explored to improve pregnancy and delivery rates per embryo transfer. While a better understanding of embryo quality could help optimize the existing “in vitro fertilization” (IVF) therapy schemes, it is essential to address the economic viability of such technologies in the healthcare setting.

**Methods:**

An Embryo-Dx economic model was constructed to assess the cost-effectiveness of 3 different IVF strategies from a payer’s perspective; it compares Embryo-Dx with single embryo transfer (SET) to elective single embryo transfer (eSET) and to double embryo transfer (DET) treatment practices.

**Results:**

The introduction of a new non-invasive embryo technology (Embryo-Dx) associated with a cost up to €460 is cost-effective compared to eSET and DET based on the cost per live birth. The model assumed that Embryo-Dx will improve ongoing pregnancy rate/realize an absolute improvement in live births of 9% in this case.

**Conclusions:**

This study shows that improved embryo diagnosis combined with SET may have the potential to reduce the cost per live birth per couple treated in IVF treatment practices. The results of this study are likely more sensitive to changes in the ongoing pregnancy rate and consequently the live birth rate than the diagnosis costs. The introduction of a validated Embryo-Dx technology will further support a move towards increased eSET procedures in IVF clinical practice and vice versa.

## Background

Increasing the efficiency of the “in vitro fertilization” (IVF) procedure by improving pregnancy/implantation rates and at the same time lowering (or avoiding) the risks of multiple gestations are the primary goals of the current assisted reproductive technology [1]. These goals require a substantially improved gamete/embryo testing and selection procedure which cannot be achieved by the traditional evaluation method based on morphological assessment. New techniques in assessing oocytes and embryo quality are currently explored to improve pregnancy and delivery rates per embryo transfer. For instance, ‘Omics’ technologies, including transcriptomics, proteomics, and metabolomics have begun providing evidence that viable oocytes/embryos possess unique molecular profiles with potential biomarkers that can be used for the developmental and/or viability selection [2]. Dynamic assessment of embryonic development by time-lapse imaging based on morphological grading as well as providing kinetic parameter presents another opportunity for optimizing embryo selection*.* A number of new non-invasive embryo viability diagnostic tests are under development to allow a rapid objective ranking of a patient’s cohort of embryos for transfer in order to improve clinical pregnancy and delivery rates per embryo transfer, thus encouraging greater uptake of single-embryo transfer (SET).

While a better understanding of embryo quality could help optimize the existing therapy schemes, it is essential to address economic viability of such technologies in the healthcare setting. As oocyte/embryo diagnostic (Embryo-Dx) procedures prepare to enter the market, health care decision makers (payers) will assess whether increases in efficacy (i.e. live births) are significant enough to justify the additional costs of the diagnostic procedure. If improved diagnostic success prevents patients from requiring additional fresh or frozen cycles it might be possible to realize a budget neutral scenario or potentially cost-savings.

The objective of this study was to assess the clinical and economic outcomes associated with non-invasive embryo diagnostics (Embry-Dx) introduction into IVF standard treatment practices. For this purpose, the cost-effectiveness of different IVF strategies (with and without Embryo-Dx) has been compared from a payer’s perspective.

### Value of non-invasive embryo technologies

The current research on non-invasive oocytes and embryo technologies comprises both morphometric and biomarker assessments. Morphometric assessment is focused on the automatization and standardization of current morphological grading procedures; i.e. an incubator plus a camera providing time-lapse images of embryo. A dynamic assessment of embryonic development (cleavage kinetics) using automated time-lapse imaging systems may have the potential to improve oocytes/embryo selection [3]. Biomarker assessment is trying to identify predictive biomarkers of oocyte/embryo viability via gene expression profiling of cumulus cells surrounding the oocyte, and proteomic and metabolic approaches in embryo culture media using quantitative real-time Polymerase Chain Reaction (PCR)-based assays microarray technologies or mass spectrometry. The development of accurate and validated tests for embryo ranking including endometrial receptivity may significantly improve non-invasive embryo quality assessment. There are expectations with these new approaches to improve on-going pregnancy rates between 5-15% (absolute increase) dependent on the methodology [4], but all of the new approaches still need to prove clinical utility through prospective randomized clinical trials. Although, considerable challenges lay ahead as effective classification systems for ranking embryos continue to be developed, there is a clear need for a reliable and non-invasive method of embryo selection to ensure that only embryos with the highest development potential are chosen for transfer thus reducing the need for multiple transfers and consequent risk of multiple births. This would support policies on elective single embryo transfer (eSET) in many countries (e.g. HEFA^a^ policy in the UK). Elective single-embryo transfer has been proposed as a strategy to reduce the risk of multiple births, which are associated with increased maternal and neonatal complications as well as increased costs to the health service. However, such eSET policies can only be applied successfully in combination with high quality embryo selection and good cryopreservation programs [5].

While a better understanding of embryo quality could help optimize the existing therapy schemes, it is essential to address economic viability of such technologies in the healthcare setting. Given the current health care environment and limited health care resources there is a need to consider the opportunity cost of decisions and to evaluate efficacy and economic consequences of different IVF strategies with and without embryo diagnostics, and hence to assess Embryo-DX technology in the health economic context. Health economic evaluations are increasingly used to support policies on reimbursement and pricing for new innovative healthcare technology, as well as to evaluate and advise on its use in clinical practice [6]. A health economic evaluation (e.g. cost-effectiveness analysis) is defined as a comparative analysis of both the cost and the health effects of two or more alternative health interventions [7]. Such an analysis makes it possible to examine whether the money that would be invested in a new intervention for a particular condition would actually be used efficiently. The net costs can be balanced with the net health effects, often expressed in quality-adjusted life-years (QALYs) and the ratio, the so called ICER (incremental cost-effectiveness ratio) between both can be assessed [8].

## Methods

### Model design

**Figure 1:**
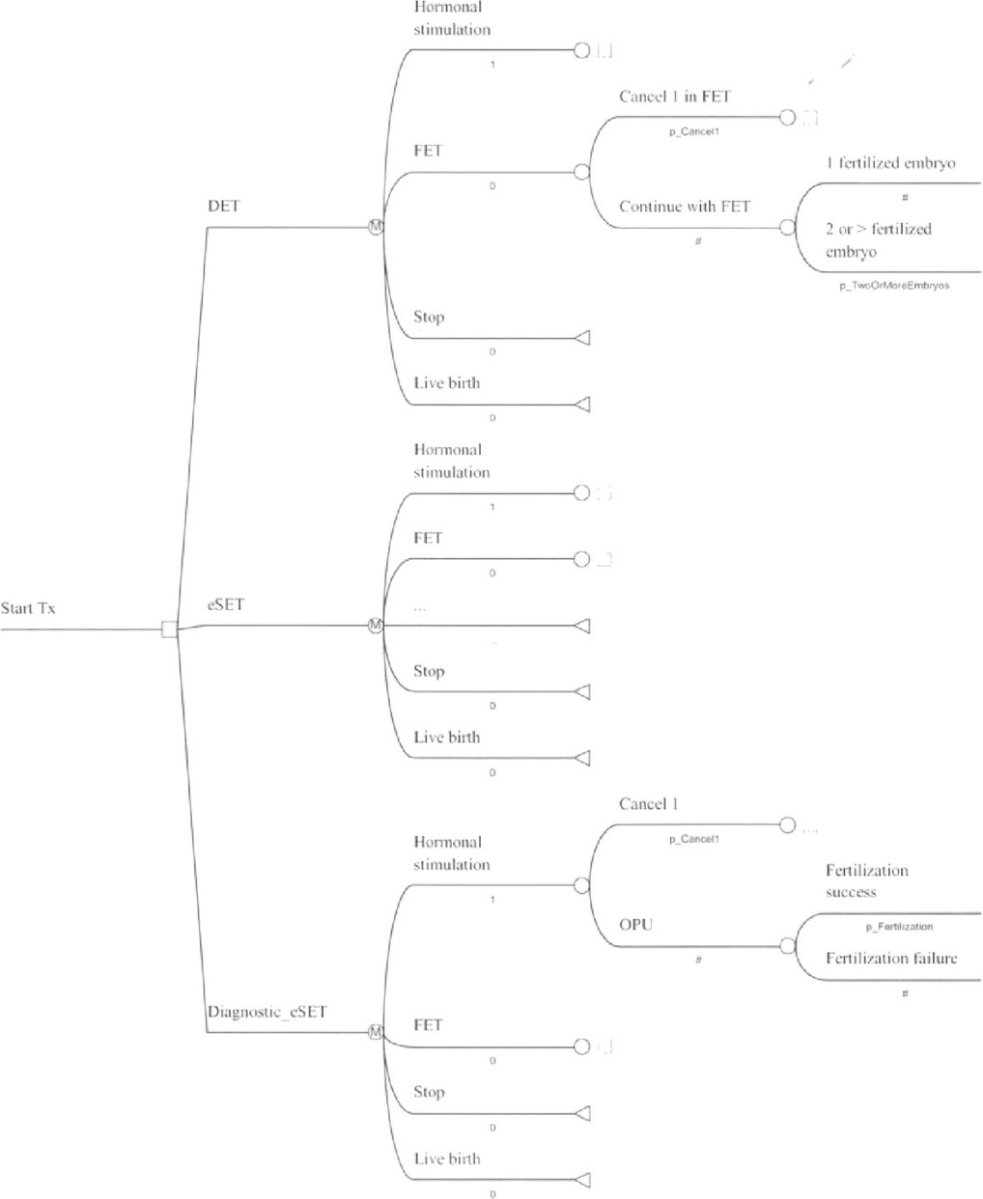
**Embryo-Dx model.**

A decision analytical Markov model (Embryo-Dx model - see Figure 1) was constructed to assess the economic consequences of 3 different IVF strategies. The Embryo-Dx model:

- Compares Embryo-Dx with single embryo transfer (Embryo-Dx/SET) to eSET and to double embryo transfer (DET) treatment practices and
- Considers a maximum of one fresh and one frozen cycle in the comparison of the different strategies regarding their costs and life birth rates.

The strategies were selected for clinical relevance. Elective single-embryo transfer has been proposed in many health care systems as a strategy to reduce the risk of multiple births, which are associated with increased maternal and neonatal complications as well as increased costs to the health service. For instance, in The Netherlands the current policy is to offer SET in good prognosis patients (i.e. young patients with a good quality embryos). On the other hand, the DET strategy of transferring two embryos into the uterus is still customary in the majority of women receiving IVF treatment, particularly in older women [9].

There have been many studies comparing the economic consequences of SET vs DET in various health care systems [10–12]. Also, several cost-effectiveness studies have shown that transferring one fresh embryo and then, if needed, using one frozen and thawed embryo may dramatically reduce the number of twin pregnancies while achieving similar cumulative pregnancy rates compared to DET in good prognosis patients [13]. A cost-effectiveness study by Fiddelers et al. [14] in The Netherlands compared seven embryo transfer strategies varying eSET, DET and standard treatment procedure. In this study clinical outcomes data came from a randomized clinical trial (RCT) performed at the University Hospital of Maastricht (Montfoort et al. [15]) where 308 couple were randomized between eSET and DET, irrespective of female age and embryo quality. The cost data were based on the Dutch healthcare system. The Embryo-DX model uses the same data sources in The Netherlands because it provided detailed data on cost and efficacy parameters including treatment costs in relation to treatment success, embryo fertilization, frozen cycles, embryo production and pregnancy rates as well as a broad range of multiple pregnancy and post –delivery cost. The results discussed here are broadly applicable to other markets, however variation in the costs may change some of the results described here.

#### Embryo-DX technology

Several technologies are currently being developed to improve embryo selection with the aim of improving live birth rates and reducing multiple pregnancy rates. For the purposes of the analysis described here we consider embryo diagnostic testing from a theoretical perspective. Therefore, the efficacy improvements discussed here are not based on any specific technology and are only used for purpose of illustration and clinical development. From an economic perspective we can assess the anticipated benefits with respect to the expected costs in order to inform decision-making.

### Data sources

#### Treatment assumptions and probabilities

The following assumptions have been used in constructing the model:

1. Patients with frozen embryos would progress to frozen embryo transfers in the second cycle.
2. Embryo diagnostics testing would not be performed in patients with only 1 viable embryo. This represents approximately 9% of patients treated in The Netherland [16].
3. The benefits of embryo diagnostics are only observed in fresh treatment cycles. For patients undergoing embryo diagnosis and progressing to frozen cycles, embryo diagnosis would have no observable benefit in frozen cycles.
4. The cost of the embryo diagnostic procedure has been included as a fixed cost irrespective of the number of embryos retrieved and evaluated.
5. With the introduction of Embryo-Dx it was assumed that DET transfer policy would not be used. This was based on expert advice that Embryo-Dx would minimize the need for DET because of the improved efficacy and the use of DET would further increase risk of multiple pregnancy.
6. Improved efficacy was accounted for by adjusting the ongoing pregnancy rate.

These assumptions have been developed in conjunction with IVF experts (see acknowledgement). The probabilities for pregnancy rates are outlined in Table 1.

**Table 1:** **Probabilities used as input for the Embryo-Dx model**

Clinical data		Source
Pregnancy rates		
After eSET (%)	21.4	RCT data (n = 308) Maastricht (Montfoort et al. 2006)
After DET (%)	40.3	RCT data (n = 308) Maastricht (Montfoort et al 2006)
After DxSET	33.4	Expert opinion

For more clinical parameter see Fiddelers et al. supplementary data; *RCT*: Randomized controlled trial.

#### Technical note to Embryo-Dx probabilities

In the Embryo-Dx model the on-going pregnancy rate for eSET and DET was 21.4% and 40.3%, respectively. Within the model we assumed that embryo diagnostics improved the ongoing pregnancy rate with SET, and that this ultimately resulted in improved live birth rates. There are several reasons why adjustments were made to the “ongoing pregnancy” rate and not to the probability of “live birth” directly. Firstly, adjusting the ongoing pregnancy rate ensures that all upstream costs in the model are accounted for. For instance, monitoring visits that occur during the ongoing pregnancy have to be considered appropriately. Secondly, because the model uses a series of probabilities, it is constrained by the numbers of people progressing through various stages of the model. If adjustments were made only to the end probability, it would be constrained by the number of people in earlier Markov stages. Therefore, much larger increases to the end probability would have been required to achieve the improved efficacy associated with Embryo-Dx in the model.

#### Costs included in model

The cost analysis was performed from a payer perspective and included direct medical costs within the health care sector. The costs were determined empirically for each couple starting IVF cycle up to 6 weeks after birth. The Embryo-Dx model included the following cost variables in the analysis: Cost of IVF treatment (hormonal stimulation, oocyte pickup, Laboratory, embryo transfer), costs of a singleton and twin pregnancy (complicated and non-complicated pregnancy), costs of delivery of a singleton and twin and costs of the period from birth until 6 weeks after birth, for the mothers as well as the children (see Table 2). All costs were based on data from the Netherlands and were converted to the index year of 2013 according to the consumer price index (CPI, 2013).

**Table 2:** **Mean costs IVF cycle until four weeks after delivery for all 308 patients included in the study**

Markov cycle	Resources included in model	Costs per couple ^1^(€) mean
IVF treatment cycle	Medication	1570
	Hospital care	325
	Ovum pick-up (OPU)	586
	Laboratory	1314
	Embryo transfer	310
	Hospital admission/Others (GP’s)	909
Pregnancy singleton (5-40 weeks)	Complications	4605
	Hospital costs: consults, ultrasound	
	No Complications	1522
	Hospital costs: consults, ultrasound/Others	
Pregnancy twin (5-40 weeks)	Complications	4605
	Hospital costs: consults, ultrasound	
	No Complications	2062
	Hospital costs: consults, ultrasound/Others	
Delivery singleton up to 6 weeks post delivery	Hospital admission and delivery	12438
	Singleton complication costs	
	Other health care costs	
Delivery twin up to 6 weeks post delivery	Hospital admission	41844
	Twin complication costs	
	Other health care costs	

^1^Cost per couple = unit price times volumes of use.

#### Base case

The model reflects a base set of assumptions for costs and efficacy associated with introducing embryo diagnosis into treatment practices. The base assumption on efficacy is an ongoing pregnancy rate of 33.4% with Embryo-Dx. This translates into an approximate 9% improvement in the live birth rate with Embryo-Dx. The cost of the Embryo-Dx included in the model was €400 per test regardless of the number of embryos that were harvested.

### Model output

The model estimates several parameters useful for medical decision-making.

**Firstly**, the model generates the cost per couple treated. This does not include only the costs of fertility treatment, but also costs associated with the proportion of people with a live birth, costs of multiples, and associated medical costs up to 6 weeks post-delivery. It was necessary to incorporate a range of costs in order to reflect the advantages of embryo diagnostics on cost savings associated with multiple pregnancies. Therefore, the cost per couple does not reflect the cost per cycle as typically described in the literature.

**Secondly**, the model calculates live birth rates based on one fresh IVF cycle and the cumulative live births following a second frozen cycle. The number of cycles was limited to one fresh and one frozen because of uncertainty regarding how embryo diagnosis would impact on treatment success beyond the first cycle.

**Thirdly**, the model generates the “cost per live birth” and “incremental cost-effectiveness ratio (ICER)” for the three interventions compared. The ICER is the extra cost for a gain in one extra live birth, when two treatments are compared. The cost per live birth and the ICER are common metrics in cost-effectiveness studies in assisted reproductive technologies (ART) and are familiar to paying audiences.

## Results

For the base case the cost and live birth rates after a single fresh cycle followed consecutively by one frozen cycle for eSET, DET and Embry-Dx SET are described in the Table 3 below. The cost per live birth for Embryo-Dx SET is the lowest compared to the other strategies. The improved live birth rate with Embrxo-Dx is still lower than the success rates achieved with DET after two cycles. The cost of a new Embryo-Dx explored in the base example was €400. The ICER of Embryo-Dx SET compared to eSET versus DET compared to eSET is lower (€15,439 versus €25,509). The ICER for DET versus Embryo-Dx SET is €52,674.

**Table 3:** **Cumulative costs and live birth rates for one fresh and one frozen cycle transferring SET, DET and Embryo-Dx SET (Base case)**

Strategy	Cost	Incremental cost	Live births	Cost per live birth	ICER vs. eS ET	ICER vs Embry Dx SET
eSET	€14,896	-	0.314	€47,439		
Embryo-Dx SET	€16,687	€1,791	0.43	€38,807	€15,439	
DET	€18,952	€2,265	0.473	€40,068	€25,509	€52,674
		*€4,056‡*				

‡Based on cost comparison with eSET.

### Sensitivity analyses

Appropriate sensitivity analyses were performed to test how sensitive the results were to changes in model parameter values for costs and clinical probabilities. A sensitivity analysis is based on the modification of the basic clinical and economic estimates of input variables over a plausible range of values to judge the effect on study results of alternative assumptions for the range of potential values for uncertain variables. Sensitivity analyses have been performed for both the cost per live birth and the ICER.

### Embryo-Dx success sensitivity analysis

**Figure 2:**
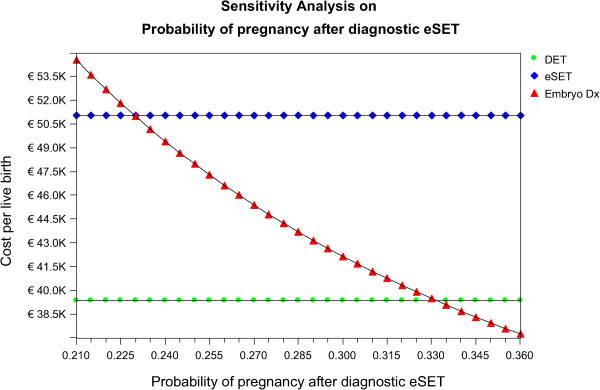
**Variation in embryo diagnosis on cost per live birth.**

As the benefit of embryo diagnostics are only observed in fresh treatment cycles the variation in costs and live birth rates are only assessed here for 1 fresh cycle of IVF. Transitions in the cost per live birth based on variations in the live birth rates with Embryo-Dx are illustrated in Figure 2. In this analysis the live birth rate is increased for Embryo-Dx SET patients while live birth rates for eSET and DET are held constant. When the ongoing pregnancy rate for Embryo-Dx is between 0.210 – 0.225 this is the least cost-effective option. However, when the ongoing pregnancy rate for Embryo-Dx is between 0.23 – 0.33 this option is more cost-effective than eSET. When the ongoing pregnancy rate reaches 0.335 Embryo-Dx becomes more cost-effective than DET (point where red line crosses green line).

### Embryo-Dx cost sensitivity analysis

Because price is an important component that influences reimbursement, a sensitivity analysis was conducted based on variations in the acquisition cost for a new Embryo-Dx. In this analysis the purchase price was varied from €200 - €600 while the base assumption for improved ongoing pregnancy rate with Embryo-Dx was held constant at 33.4% as the price of the test was varied.

**Figure 3:**
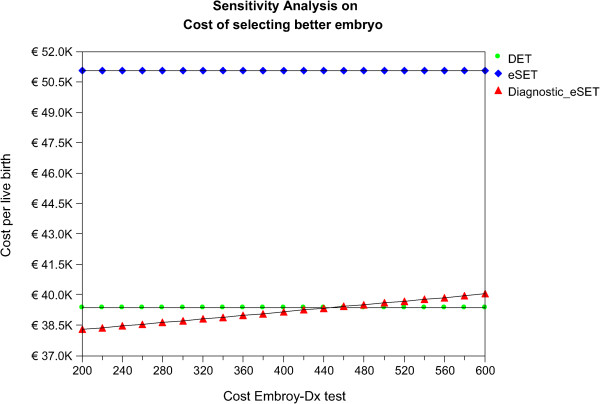
**Variation in cost of Embryo-Dx on cost per live birth.**

The analysis shows that at a price of €200 - €460 the Embryo-Dx results in the lowest cost per live birth compared to eSET and DET. From €480 - €600 the cost per live birth with Embryo-Dx SET is lower than eSET, and slightly higher than DET (Figure 3).

In addition, we conducted extensive one-way sensitivity analyses on the ICER for key input parameters, which may have an impact on the outcome of the cost-effectiveness analysis when analysis is performed for 2 cycles. The variation of the values was based on plus and minus 20% of the base case value. The sensitivity analyses (Table 4) show that pregnancy rate to DET, is most sensitivity clinical parameter with ICER ranging from €35,438 to €22,235 for comparison between DET versus eSET. The cost for embryo transfer is most sensitive economic parameter with ICER ranging from €50,314 to €54,736 for comparison DET versus Diagnostic eSET.

**Table 4:** **Sensitivity- analysis**

Clinical probabilities	Range*				
p_Pregnancy_cSET (mean:0.091)	Input value		ICER		Difference
Diagnostic_eSET versus eSET	0.073	0.109	€15,481	€15,473	€8
DET versus Diagnostic eSET			€52,447	€52,603	€156
DET versus eSET			€25,553	€25,465	€88
p_Pregnancy_DET (mean:0.406)	Input value		ICER		Difference
Diagnostic_eSET versus eSET	0.323	0.4843	€15,477	€15,477	€0
DET versus Diagnostic eSET			Dominated**	€28,826	> €28,826
DET versus eSET			€35,438	€22,235	€13,202
p_Pregnancy_eSET (mean:0.214)	Input value		ICER		Difference
Diagnostic_eSET versus eSET	0.1714	0.2572	€17,995	€20,750	€2,755
DET versus Diagnostic eSET			€39,559	€39,559	€0
DET versus eSET			€24,256	€29,352	€5,096
**Costs**					
c_diagnostic tests (mean: €400)	Input value		ICER	Difference	Input value
Diagnostic_eSET versus eSET	320	480	€14,705	€16,249	€1,544
DET versus Diagnostic eSET			€54,596	€50,454	€4,142
DET versus eSET			€25,509	€25,509	€0
c_IVF_Medication (mean: €1570)	Input value		ICER		Difference
Diagnostic_eSET versus eSET	€1,256	€1,884	€15,705	€15,249	€456
DET versus Diagnostic eSET			€52,966	€52,085	€881
DET versus eSET			€25,792	€25,220	€572
c_IVF_HospCare (mean: €325)	Input value		ICER		Difference
Diagnostic_eSET versus eSET	€260	€390	€15,524	€15,430	€94
DET versus Diagnostic eSET			€52,616	€52,434	€182
DET versus eSET			€25,566	€25,447	€119
c_IVF_Laboratory (mean:€1314)	Input value		ICER		Difference
Diagnostic_eSET versus eSET	€1,051	€1,577	€15,596	€15,358	€238
DET versus Diagnostic eSET			€52,328	€52,723	€395
DET versus eSET			€25,541	€25,478	€63
c_IVF_EmbryoTransfer (mean:310)	Input value		ICER		Difference
Diagnostic_eSET versus eSET	€248	€372	€15,522	€15,432	€90
DET versus Diagnostic eSET			€50,314	€54,736	€4,422
DET versus eSET			€24,943	€26,075	€1,132

*Range: probabilities: plus/minus 20% but between 0 and 1.

**Diagnostic eSET is more effective and cost saving versus DET.

The cost for diagnostic tests is not a very sensitive economic parameter with ICER ranging from €54,596 to €50,454 for comparison DET versus Diagnostic eSET.

The results of the sensitivity analyses show that the outcomes of the model are robust to the uncertainty in the input parameters of the model. Therefore the concept, which has been presented, is not affected by huge uncertainty of the underlying model, and therefore the model suits for the purpose of illustration of the concept.

## Discussion

New techniques in assessing oocytes and embryo quality are currently explored to improve clinical pregnancy and delivery rates per embryo transfer. The identification of high-quality oocytes and embryos using objective non-invasive technologies could help optimize existing IVF therapy schemes, thus encouraging greater uptake of single-embryo transfer. However, translating new innovative techniques (both morphometric and biomarker assessments) into clinical practice awaits evidence of their clinical utility. Good - quality studies of these techniques are needed and results need to be validated in clinical settings to determine their potential clinical and economic application. In addition, successful embryo implantation will require endometrial receptivity and an adequate bi-directional communication between the blastocyst and endometrium [17].

The Embryo-Dx model showed that under a set of base assumptions the introduction of Embryo-Dx exam into IVF is cost-effective compared to eSET. Based on a price of €400 per embryo diagnosis, the cost per live birth for Embryo-Dx is the lowest (€38,807) compared to eSET and DET. The ICER of Embryo-Dx SET compared to eSET is €15,439 for an extra live birth. DET is more effective but also more costly compared to Embryo-Dx with an ICER around €52,000 for an extra live birth. This situation may be different if long-term cost aspects by avoiding of high cost multiple pregnancies triggered by DET will be considered from a broader societal perspective. However, it depends on payers’ willingness to pay whether such new technologies will be applied in clinical practice. Although no agreement exists on an appropriate ceiling ration for one extra live birth, as opposed to the ceilings ratio for a QALY, a ceiling ration of about €15,000 (vs eSET) for Embryo-Dx testing in this study seems low. Also, assisted reproductive treatments present difficulties for the QALY approach, as the main outcome of an IVF treatment is a live birth. While QALYs are intended to capture improvements in health among patients, they are not appropriate for placing a value on additional lives which is the intended purpose of assisted reproduction [18]. Furthermore, a comprehensive economic value assessment of new technologies may also require a budget impact analysis (BIA) to estimate the impact of the new intervention on short- or longer-term annual healthcare budgets. Especially local budget holders are interested to evaluate the economic impact of using such new diagnostic testing with focus on budget impact to ensure getting sufficient value and cost offsets.

Decision making between SET and DET depends not only on ongoing pregnancy rates and twin pregnancy rates, but also on several other factors such as age (prognostic indicator), patients’ preference and the health care system in a particular country [19]. For instance, the extent of reimbursement/coverage of the cost of new technologies will influence the acceptance of Embryo-Dx in many markets. Current diagnostic reimbursement policies in the US and many EU countries do not necessarily support the development of high-value molecular tests, as reimbursement of these tests has typically been based on cost, not on value (or potential) value [20]. Funding is restricted to hospital/clinical budget and third party payers in these markets are not willing to cover higher priced molecular diagnostics outside the standard procedures /DRG’s (diagnostic related groups). Often, flexible innovative payment approaches outside existing reimbursement schemes are needed to realize the benefits of these technologies on a case-by case basis.

The economic model presented in this analysis has some limitations. First, with respect to the scope of the Embryo-DX model the cost-effectiveness analysis only covers short-term (1-year) cost and health outcomes from a payers’ perspective not including the long-term costs associated with children born as a result of a multiple pregnancy. Currently, an on-going TwinSing study (Maastricht University Medical Centre) [21] is investigating the long-term costs and outcomes of IVF singletons and twins and it may be interesting to apply such a long-term perspective to an Embryo-diagnosis model. Second, adding Embryo-Dx to current IVF treatment practice will increase complexity and complicates value assessment, including uncertainties about diagnostic characteristics (e.g. test performance) as well as gaps in the evidence supporting clinical utility.

## Conclusion

Within the limitations of this model, the results of this study show that improved embryo diagnosis will likely reduce the cost per live births per couple treated in IVF treatment practices, although this conclusion is price sensitive. The cost per live birth for Embryo-Dx is the lowest (€38,807) compared to eSET and DET, offsetting assumed diagnosis cost of €400 in this analysis and reflecting an improved cumulative delivery rate. The results of this study are likely more sensitive to changes in the ongoing pregnancy rate and consequently the live birth rate than the diagnosis costs. The introduction of a validated Embryo-Dx technology will further support a move towards increased eSET procedures in IVF clinical practice and vice versa. It also may trigger healthcare coverage and reimbursement policies addressing appropriate DRG’s and value-based diagnostics assessment for assisted reproductive technologies (ART). Finally, this assessment may outline pricing opportunities/limits for the industry to develop certain embryo diagnostic testing products for commercialization.

## Endnote

^a^HEFA/UK: Human Embryology and Fertilization Authority.
